# High Mortality Risk in Hypoglycemic and Dysglycemic Children Admitted at a Referral Hospital in a Non Malaria Tropical Setting of a Low Income Country

**DOI:** 10.1371/journal.pone.0150076

**Published:** 2016-02-24

**Authors:** Hubert Barennes, Eng Sayavong, Eric Pussard

**Affiliations:** 1 Institut de la Francophonie pour la Médecine Tropicale, Vientiane, Lao PDR; 2 Agence Nationale de Recherche sur le VIH et les Hépatites, Phnom Penh, Cambodia; 3 Epidemiologie-Biostatistique, ISPED, Centre INSERM U897, Bordeaux University, F-Bordeaux, France; 4 Epidemiology Unit, Pasteur Institute, Phnom Penh, Cambodia; 5 Génétique Moléculaire, Pharmacogénétique et Hormonologie, Kremlin Bicêtre University Hospital, Paris, France; University of Texas Health Science Center at San Antonio, UNITED STATES

## Abstract

**Introduction:**

Hypoglycemia is a recognized feature of severe malaria but its diagnosis and management remain problematic in resource-limited settings. There is limited data on the burden and prognosis associated with glycemia dysregulation in non-neonate children in non-malaria areas. We prospectively assessed the abnormal blood glucose prevalence and the outcome and risk factors of deaths in critically ill children admitted to a national referral hospital in Laos.

**Methods:**

Consecutive children (1 month-15 years) admitted to the pediatric ward of Mahosot hospital, were categorized using the integrated management of childhood illness (IMCI). Blood glucose was assessed once on admission through a finger prick using a bedside glucometer. Glycemia levels: hypoglycemia: < 2.2 mmol/L (< 40 mg⁄ dl), low glycemia: 2.2–4.4 mmol/L (40–79 mg⁄ dl), euglycemia: 4.4–8.3 mmol/L (80–149 mg⁄ dl), and hyperglycemia: > 8.3 mmol/L (≥150 mg⁄ dl), were related to the IMCI algorithm and case fatality using univariate and multivariate analysis.

**Results:**

Of 350 children, 62.2% (n = 218) were severely ill and 49.1% (n = 172) had at least one IMCI danger sign. A total of 15 (4.2%, 95%CI: 2.4–6.9) had hypoglycemia, 99 (28.2%, 95%CI: 23.6–33.3) low glycemia, 201 (57.4%, 95% CI: 52.0–62.6) euglycemia and 35 (10.0%, 95% CI: 7.0–13.6) hyperglycemia. Hypoglycemia was associated with longer fasting (p = 0.001) and limited treatment before admission (p = 0.09). Hypoglycemia and hyperglycemia were associated with hypoxemia (SaO2) (p = 0.001). A total of 21 (6.0%) of the children died: 66.6% with hypoglycemic, 6.0% with low glycemic, 5.7% with hyperglycemic and 1.4% with euglycemic groups. A total of 9 (2.5%) deaths occurred during the first 24 hours of admission and 5 (1.7%) within 3 days of hospital discharge. Compared to euglycemic children, hypoglycemic and low glycemic children had a higher rate of early death (20%, p<0.001 and 5%, p = 0.008; respectively). They also had a higher risk of death (OR: 132; 95%CI: 29.0–596.5; p = 0.001; and OR: 4.2; 95%CI: 1.1–15.6; p = 0.02; respectively). In multivariate analyses, hypoglycemia (OR: 197; 95%CI: 33–1173.9), hypoxemia (OR: 5.3; 95%CI: 1.4–20), presence of hepatomegaly (OR: 8.7; 95%CI: 2.0–37.6) and having an illiterate mother (OR: 25.9; 95%CI: 4.2–160.6) were associated with increased risk of death.

**Conclusion:**

Hypoglycemia is linked with a high risk of mortality for children in non malaria tropical settings. Blood sugar should be monitored and treatment provided for sick children, especially with danger signs and prolonged fasting. Further evaluations of intervention using thresholds including low glycemia is recommended in resource-limited settings. Research is also needed to determine the significance, prognosis and care of hyperglycemia.

## Background

Both hypo and hyperglycemia occur frequently in sick children and have been associated with increased risk of deaths in pediatrics units [1,2]. Neonates and young children are particularly susceptible to hypoglycemia leading to the well recognized long term sequelae especially with prolonged fasting [3–7]. While this dangerous problem is greatly recognized in western countries, reports have only started to emerge about neonatal hypoglycemia in developing countries [8–12].

Young children have a limited tolerance for fasting [1]. Hypoglycemia in non-neonate children is independently associated with poor outcomes in the tropics [13–19]. It is a well recognized feature and predictor of death in severe malaria [3,20–29] and was often aggravated by quinine treatment [22,24,30–33]. The iatrogenic hyperinsulinemia after quinine treatment is expected to progressively disappear with the switch to artesunate combination therapy to treat malaria [34]. The management of hypoglycemia remains one of the leading priorities to decrease the child-malaria fatality rate [35].

Hypoglycemia is also related with poorer prognosis in infections [17,36], diarrhea and dehydration [2], malnutrition [15,37] and intoxications [38]. In resource limited settings, hypoglycemia may be aggravated by local conditions such as: poor nutritional status; delay in admittance to hospital and lack of diagnostic facilities [13,19].

The diagnosis and management of hypoglycemia and low glycemia raises further concerns. There is a considerable variability in the thresholds used to define hypoglycemia in non neonate ill children among studies and guidelines Table 1 [39]. These definitions are based mainly on expert opinion, and are still not definite [14]. Firstly, severe hypoglycemia in critically ill patients was arbitrarily defined in most studies as when blood glucose fell below 2·2 mmol/L (40 mg/dl) on at least one occasion. This cut-off was used in severe malaria many years ago [40,41] and recommended for emergency intervention [42]. It is still recommended for severe malaria [43]. Currently, outside the neonatal period, hypoglycemia is defined by blood glucose <45 mg/dl (<2.5 mmol/L) in a well nourished child or <54 mg/dl (3 mmol/L) in a malnourished child [42]. International paediatric guidelines recognize blood glucose below 3 mmol/L in children with severe disease as a therapeutic indication for treatment [44]. In surveys, the prevalence of hypoglycemia has been infrequently assessed using different thresholds in critically ill non-neonate children admitted to hospitals in the tropics [1,12–13,15,17–18,22,28,45–48].

**Table 1:** Cut-off values recommended or used for hypoglycemia and low glycemia in non neonate children in the tropics.

Hypoglycemia		Low glycemia	Hyperglycemia	Context	Reference
Threshold		Threshold^a^	Threshold^a^		
(mg ⁄ dl)	(mmol ⁄ l)	(mmol ⁄ l)	(mmol ⁄ l)		
< 40	< 2.2	-	-	Severe malaria	White et al., 1987 [40]
			-		Marsh et al., 1995 [20]
			-		English et al., 1998 [22]
			-		WHO, 2000 [41]
			-		WHO, 2015 [43]
< 40	< 2.2	**-**	> 10.0	On admission	Osier et al., 2003 [12]
			-	Humanitarian emergencies	WHO, 2008 [42]
< 40	< 2.2	2.2–4.4	≥ 8.3	Severe malaria	Willcox et al., 2010 [28]^b^
< 40	< 2.2	-	-	Severe malaria	Jallow et al., 2012 [21]
< 40	< 2.2	2.2–4.4	≥ 8.3	Non malaria	Sambany et al.; 2013[13]^b^
< 40	< 2.2	2.2–3.9	> 10.0	Adult and children	Mesotten et al., 2015 [47]^c^
< 45	< 2.5	-		Well-nourished and severe infection	WHO, 2013 [42]	
< 45	<2.5	2.5–5.0		Febrile illness	Nadjm et al., 2013 [17]
< 47	< 2,6	-		Malaria	Onyiriuka, 2012 [3]
< 54	< 3.0	-		Severe malnutrition	WHO, 2013, [42]
		-		Young children	
		-		Severe disease	Group AL, 2004 [44]
< 60	< 3.3			Severe malaria	Graz et al., 2008 [46]
< 40	< 2.2	-		Severe malaria	Ogetii et al., 2010 [32]
< 45	< 2.5	-			Ogetii et al., 2010 [32]	
< 60	< 3.0	-			Ogetii et al., 2010 [32]	

^a^ To convert to mg/dl: multiple the result by 18

^b^Same threshold were used in the current study in Lao PDR

^c^ In western countries

Secondly, we and others questioned the 2.2 mmol/L (40 mg/dl) threshold, defined a low glycemia range of 2.2–4.4 mmol/L (40–79 mg⁄dl) [13,28] and showed that low glycemia was also independently associated with a higher risk of death among 481 children in Mali [28]. We suggested including low glycemia as a new threshold for intervention in tropical countries. This proposal offers the benefit of early intervention and prevention to avoid further aggravation related to blood glucose metabolism in severely ill children. This was further supported by two surveys in malaria areas: one large retrospective analysis of glucose measurements of 23805 children aged 1–59 months admitted in Kilifi hospital Kenya in a high malaria transmission region and a prospective survey among 3319 children with febrile disease in Tanzania [14,17]. Cut-offs of 4 mmol/L (72 mg/dl) and 5 mmol/L (90 mg/dl) were predictors of mortality in the first and second survey, respectively.

On the other hand, hypoglycemia is frequently asymptomatic in severely ill children, so health staff may be unaware of this common problem. Hypoglycemia can mimic various clinical signs or aggravate a more obvious disease. Systematic screening for hypoglycemia in sick children is now recommended by WHO [42] and point of care tools are becoming more available in referral hospitals [1,48]. When diagnostic tools are not available, a systematic treatment of assumed hypoglycemia is recommended for children with severe malnutrition [42].

The integrated management of childhood illness (IMCI) algorithm is a tool that helps to triage the severity disease in children according to the level of health facilities and of emergency [49]. We previously showed that hypoglycemia was associated with both IMCI-classified severe disease and poor prognosis, but the IMCI algorithm was a tool of low specificity for detecting glycemia disturbances [13]. We also showed the association of both hypoglycemia and hyperglycemia with increased risk of deaths and the lower IMCI status of children with low glycemia [13].

Thus, to fill the gap of limited data on glycemia disorders in non neonate children from low income settings where malaria is not a major issue, we prospectively assessed the prevalence, outcome and risk factors of death in critically ill children admitted to a national referral hospital in Laos.

## Methods

We used a similar procedure as our previous study in Madagascar and adapted it to the Lao hospital [13].

### Ethics statement

This study was part of a master’s study by “Institut Francophone pour la Médecine Tropicale” (IFMT, Vientiane, Laos). An ethical clearance for the study was requested and approved by the Lao Medical Ethics Committee and the study was conducted with the agreement of the Lao health authorities. Parents/guardians were informed about the study in Lao language and given an information sheet describing the study. Children were included if their parents/guardians had given written informed consent. The study was performed in accordance with the Declaration of Helsinki [50].

### Study setting site

The study was conducted in the pediatric department of Mahosot Hospital, the main pediatric institution, in Vientiane (810,000 people), the capital of Laos, between March and July 2011. In Laos (Population: 6,803,699 people with 34.8% under 15 years, literacy level: 73%) 22% of the population lives below the poverty level of USD 1.25 per day [13,51]. The infant mortality is 55.4 deaths per 1000 live births and 31% of children below the age of five years are underweight. Malaria is restricted to the south, bordering Cambodia.

### Study site, patients and clinical procedures

The pediatric department of Mahosot Hospital (75 beds) admits on average 3000 children per year. Patients (or their parents) have to pay hospital fees and buy all necessary medical supplies (i.e. syringes and infusion sets) and prescribed medications from the pharmacy at the hospital before any treatment can begin. Incoming children are usually evaluated at the admissions unit, where a team of doctors and nurses are on duty around the clock. The IMCI algorithm is used to triage the severity of children and determine who will be admitted to the ward, referred to peripheral units, or treated and sent home. A few patients directly access the pediatric wards.

### Participants

All consecutive children (1 month—15 years) admitted to the Mahosot pediatric department during the investigators’ duty hours were eligible for the study. Emergency cases were included only if inclusion would not cause delays in their treatment. Children with known diabetes, hemophilia or history of neonatal hypoglycemia were excluded as well as children who had been previously enrolled in the study. Investigators were on duty for 24 hours, then given a 24-hour break.

### Clinical evaluation

The medical history and examination findings were recorded in a standardized pre-tested clinical form. This form recorded demographic data, disease history and previous treatment. Children were classified according to the algorithm of IMCI including the general danger signs: serious illness; signs of moderate to severe dehydration; pneumonia and nutritional status. Time and status at discharge were recorded from the hospital forms.

Children diagnosed with hypoglycemia were treated with intravenous bolus administration of 5 ml/kg of 10% dextrose, followed by dextrose infusion. The treatment for their baseline disease was started as early as possible. Children warranting hospitalization were sent to the pediatric ward for further treatment according to hospital guidelines.

In-hospital case fatalities were recorded prospectively for all patients admitted during the study period. Patients were discharged when the medical team considered them fully recovered. Children with extremely severe status, who were discharged according to the wishes of their parents and were not expected to survive, were recorded as additional case fatalities. True case fatality was confirmed during a follow up telephone call conducted three days after discharge for all children. This procedure was new compared to the Madagascar study design.

Children were weighed with 100g precision and measured (length below 2 years, height above) with 1mm precision. Nutritional Z-scores were calculated for children under the age of 5 years, using WHO software for anthropometrical Z scores. Malnutrition was defined as moderate or severe if one of the Z-scores was below -2 or -3 SD, respectively.

Level of consciousness was evaluated using the routine scoring in the ward: Blantyre Scale for children under 5 years and the Glasgow Coma Score for those over. The level of hypoxemia was determined using a pulse oximeter.

### Definitions

Severe illness was defined according to IMCI standards: presence of any general danger signs; inability to drink or drinking poorly; vomiting; convulsions; lethargy or unconsciousness; pneumonia or severe pneumonia; severe dehydration; some dehydration; persistent diarrhea; severe persistent diarrhea; very severe febrile disease; and severe malnutrition.

Children were categorized into four groups according their blood glucose levels, as previously reported: hypoglycemia: <2.2 mmol/L (<40 mg⁄ dl); low glycemia: 2.2–4.4 mmol/L (40–79 mg⁄ dl) [13,28,48]; euglycemia: 4.4–8.3 mmol/L (80–149 mg⁄ dl); hyperglycemia: over 8.3 mmol/L (≥150 mg⁄ dl) [13,28,52].

Abnormal blood glucose was defined as any of the 3 categories which differ from normoglycemia.

### Blood glucose measurements

As soon as it was possible after arrival and after informed consent, 0.6 μL of blood was collected by investigators through a finger prick to measure the blood glucose concentration (Accu-Chek® Performa glucometers from ROCHE Laboratories, with a sensitivity limit of 1 mmol/L according to the manufacturer). After every twenty-fifth measurement, a quality control by Accu-Chek® was performed. Blood glucose concentrations were recorded in mmol/L (conversion to mg⁄dl by multiplying by a factor of 18).

We also systematically checked the blood glucose concentrations by intravenous sampling in 1 child out of 3 at the hospital laboratory.

### Outcome

The main outcome was the prevalence of each category of abnormal blood glucose. Secondary outcomes were IMCI status (disease severity), proportion of deaths within 24 hours and 3 days after discharge.

### Statistical analysis

Data was entered in EpiData freeware (S1 Dataset). All records were cross-checked with the original data sheets. Analyses were carried out with STATA, Version 8 (Stata Corporation, College Station, TX, USA).

Chi squared or Fisher’s exact tests were used to compare categorical variables as appropriate. Data distribution was graphically evaluated using the kernel density estimate and eventually tested with the Skewness and Kurtosis test and the Shapiro-Wilk test for normality. Kruskal-Wallis and Wilcoxon tests were used for non-normally distributed variables. Odds ratios were calculated with exact confidence intervals. We considered p < 0.05 as statistically significant.

Children were classified into four glycemia categories (hypoglycemia, low glycemia, normoglycemia and hyperglycemia) and into 2 IMCI categories (severe, non-severe). The four glycemia categories were globally compared and then each group with abnormal blood level was eventually compared to the euglycemia group. We investigated associations related to case fatality in models, separately. The association between admission blood glucose level and the IMCI severity level were investigated using univariate analysis according to: child’s gender; age (less or more than 3 years); duration of hospitalization; IMCI severity of illness; associated severe illness; residential area; mother’s age; education; occupation; and daily expense on food (used as an estimated proxy for poverty). Variables with a p value below 0.2 for mortality were then fitted into a multivariate logistic regression model with backward step-by-step analyses using odds ratios to evaluate the factors associated with child in-patient mortality. Only final significative variables are presented.We have attempted to report the study according to the STROBE guidelines [53].

## Results

**Fig 1:**
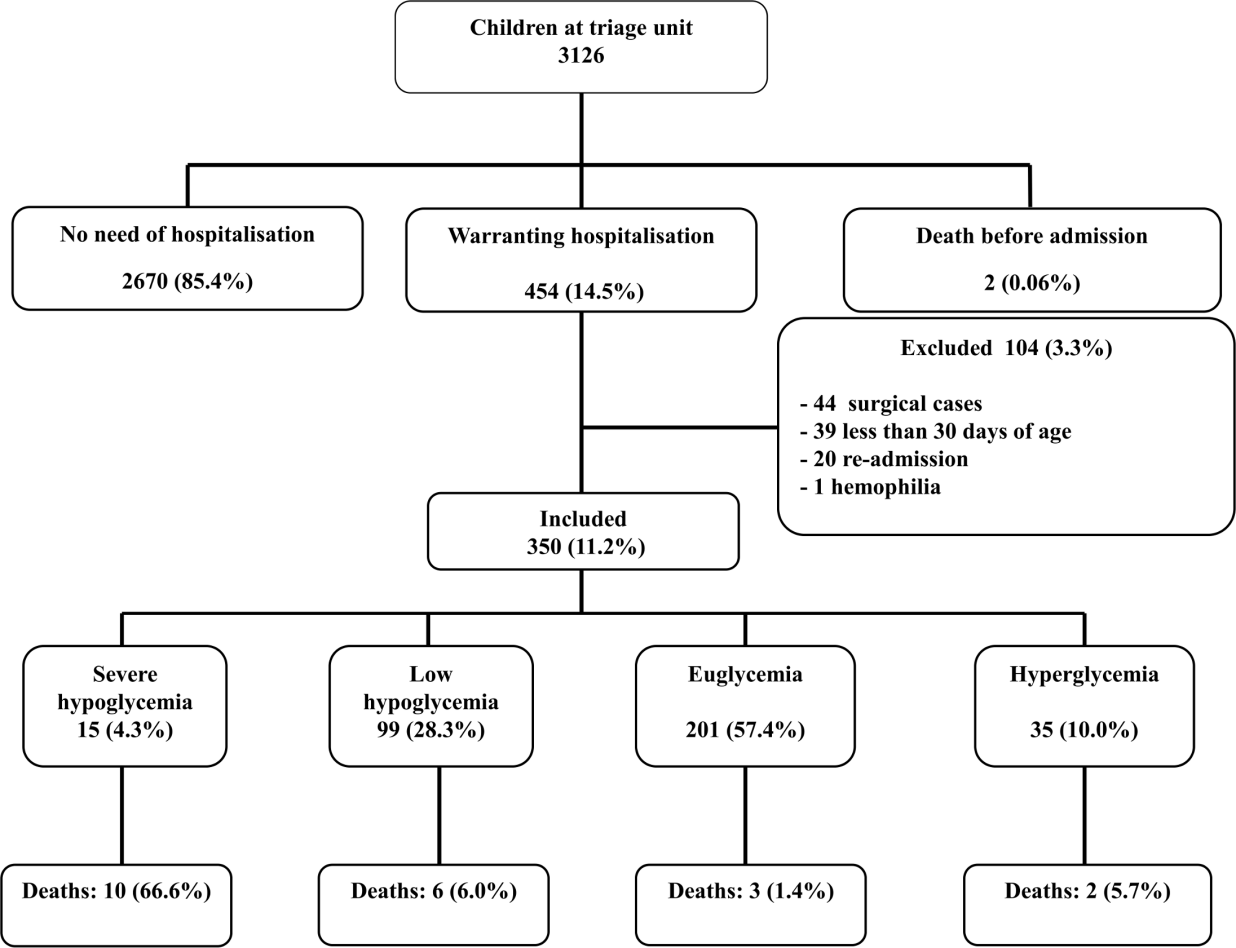
Enrollment flow chart of children on admission at Mahosot pediatric ward.

When investigators were on duty, a total of 3126 children attended the emergency unit, two died before hospitalization (0.06%) and 454 (14.5%) were admitted to the pediatric ward. One hundred and four (3.3%) were excluded leaving 350 (11.1%) children for the analyses. Reasons are shown Fig 1. A total of 44 (9.6%) surgical cases were not included in order not to interfere with clinical care. The others did not comply with the inclusion criteria. There were no other causes of exclusion.

The socio-demographic characteristics of the 350 children, according to their blood glucose at baseline are shown Table 2. Sex ratio (M/F) was 1/1.5 and the mean age 33.1 months (IQR: 9.4–100.5). The median time of admission was 2 pm and 28% of children were admitted at night. The children’s medical characteristics according to IMCI classification are summarized Table 3.

**Table 2:** Socio-demographic characteristics of sick children admitted to Vientiane hospital and their families.

	Hypoglycemia	Low glycemia	Euglycemia	Hyperglycemia	P	Total
	n = 15 (4.2%)	n = 99 (28.2%)	n = 201 (57.4%)	n = 35 (10.0%)		n = 350 (100%)
Median age (month)	31.7 [8.2–77.5]^a^	36.5 [13.8–102]	40 [9.1–101.5]	10.9 [3.2–51.6]^a^	0.01	33.1 [9.4–100.5]
Females	13 (86.6)	53 (53.5)	126 (62.6)	20 (57.1)	0.07	212 (60.5)
Urban residence	3 (20.0)	33 (33.3)	78 (38.8)	11 (31.4)	0.39	125 (35.7)
Number in the household	4 [4–7]	4 [3–5]	5 [4–6]	5 [3–6]	0.28	4[4–6]
**Mothers**						
Median age (year)	25 [22–33]^a^	29 [25–35]	30 [26–36]	28 [25–31]	0.08	29 [25–35]
**Education**						
Illiterate	1 (6.6)	3 (3.0)	6 (2.9)	1 (2.8)	0.26	11 (3.1)
Primary	7 (46.6)	21 (21.2)	59 (29.3)	12 (34.2)		99 (28.2)
Secondary	6 (40.0)	43 (43.4)	72 (35.8)	14 (40.0)		135 (38.5)
High-school	1 (6.6)^a^	32 (32.3)	64 (31.8)	8 (22.8)		105 (30.0)
**Marital status**						
Single	0	4 (4.0)	9 (4.4)	2 (5.7)		15 (4.2)
Married	15 (100.0)	95 (95.9)	192 (95.5)	33 (94.2)	0.83	335 (95.7)
**Economic level**						
Family income (US.dollars)(yr)	375 [250–625]^a^	750 [375–15,000]	750 [500–15,000]	625 [375–1000]	0.04	750 [437–15,000]
Daily budget (meal) (US. dollars)	2.5 [2.5–5.0]^b^	6.25 [3.75–7.5]	6.25 [3.75–7.5]	5 [3.75–6.25]	0.005	6.25 [3.75–7.5]
Current debts	8 (53.3)^b^	23 (23.2)	34 (16.9)	8 (22.8)	0.01	73 (20.8)
Poor	13 (86.6)^a^	61 (61.6)	119 (59.2)	21 (60.0)		214 (61.1)

Numbers and (percentages). Median and (interquartile range).

^a^p ≤0.05 when compared to euglycemia

^b^p ≤ 0.001 when compared to euglycemia (details are provided in the text).

**Table 3:** Medical characteristics of children according to IMCI algorithm^a^.

	Hypoglycemia	Low glycemia	Euglycemia	Hyperglycemia	P	Total
	n = 15 (4.2%)	n = 99 (28.2%)	n = 201 (57.4%)	n = 35 (10.0%)		n = 350 (100%)
Glycemia (mmol/L)	1.9 [1.7–2.0]	3.8 [3.7–3.9]	5.3 [5.1–5.5]	9.6 [8.9–10.3]	<0.001	5.2 [5.0–5.4]
Duration of prior illness (days)	5 [4–9]	5 [3–7]	4 [3–8]	5 [3–9]	0.8	5 [3–8]
Had treatment before admission	11 (73.3)	79 (79.8)	164 (81.5)	22 (62.8)^a^	0.09	276 (78.8)
Daytime admission (6am-6pm)	9 (60.0)^a^	70 (70.7)	151 (75.1)	22 (62.8)	0.31	252 (72.0%)
Duration of fasting (hours)	13 [1–19]^c^	3 [2–5]	2 [1–3]	2 [0–3]	0.001	2.5 [1–4]
**Anthropometrics measurements**						
Weight (kg)	12.5 [8.7–20.0]	14.7 [12.6–16.8]	15.3 [13.9–16.6]	11.1 [8.2–14.1]^a^	0.01	14.6 [13.5–15.6]
Height (cm)	84.5 [71.7–99.6]	91.4 [87.0–96.0]	91.0 [87.4–94.8]	91.0 [87.4–94.8]	0.001	89.2 [86.5–92.0]
**Vital signs**					
Temperature (°C)	38.0 (37.4–38.5)	37.6 (37.4–37.8)	37.6 (37.4–37.7)	37.8 (37.4–38.1)	0.39	37.6 (37.5–37.7)
Respiratory frequency	41.2 [33.8–48.6]^a^	30.8 [28.7–32.9]	34.3 [32.2–36.4]	51.7 (45.3–58.0)^c^	<0.001	35.4 (33.7–37.0)
**IMCI criteria**^a^						
**General danger signs**	14 (93.3)^a^	61 (61.6)	82 (40.7)	15 (42.8) ^c^	<0.001	172 (49.1)
-Unable to drink or to breastfeed	7 (46.6)^c^	10 (10.1)^c^	3 (1.4)	2 (5.7)	0.001	22 (6.2)
-Vomiting	11 (73.3)^a^	54 (54.5)^a^	72 (35.8)	11 (31.4)	0.001	148 (42.2)
-Convulsions	6 (40.0)^c^	8 (8.0)	15 (7.4)	4 (11.4)	0.001	33 (9.4)
-Lethargy or unconsciousness	11 (73.3)	8 (8.0)	10 (4.9)	4 (11.4)	0.001	33 (9.4)
**Severe IMCI**^a^	14 (93.3)^a^	68 (68.8)	124 (61.6)	32 (91.4)^c^	0.47	218 (62.2)
**Cough and respiratory difficulties**						
-Severe pneumonia	2 (13.3)	4 (4.0)^a^	34 (16.9)	21 (60.0)^c^	<0.001	61 (17.4)
-Pneumonia	0	2 (2.0)	6 (2.9)	0	0.001	8 (2.2)
**Diarrhea**						
-Acute diarrhea	6 (40.0)^b^	31(31.3)^c^	23 (11.4)	2 (5.7)	<0.001	62 (17.7)
-Severe dehydration	5 (33.3)^c^	7 (7.0)^c^	0	0	<0.001	12 (3.4)
-Moderate dehydration	2 (13.3)	25 (25.2)^c^	16 (7.9)	1 (2.8)	0.001	44 (12.5)
**Fever**						
-Very severe febrile disease	2 (13.3)^b^	50 (50.5)	97 (48.2)	12 (34.2)	0.02	161 (46.0)
**Ear disorder**						
-Mastoiditis	0	0	1 (0.5)	1 (2.8)	0.25	2 (0.5)
-Chronic ear infection	1 (6.6)	1 (1.0)	8 (3.9)	1 (2.8)	0.32	11 (3.1)
**Nutritional status**						
-Severe malnutrition^d^	4 (26.6)	11 (11.1)	22 (10.9)	9 (25.7)^b^	0.02	46 (13.1)
-Moderate malnutrition	1(6.6)	9 (9.0)	25 (12.4)	4 (11.4)	0.8	39 (11.1)
**Severe Anemia**	2 (13.3)	9 (9.0)	13 (6.4)	3 (8.5)	0.19	27 (7.7)
**Immunization status**						
-No immunization	3 (20.0)^b^	12 (12.1)	18 (8.9)	7 (20.0)	0.18	40 (11.4)
-Incomplete immunization	4 (26.6)	16 (16.1)	25 (12.4)	4 (11.4)	0.15	49 (14.0)
**SaO**_**2**_ **(%)**	91.1 [86.4–96.9]^a^	97.3 [96.5–98.1]	96.2 [95.4–97.0]	86.1 [82.2–90.2]^c^	0.001	95.1[94.3–95.9]
**Hemoglobin (g/dl)**	9.8[7.9–11.7]	8.4 [7.6–9.1]	9.1 [8.7–9.6]	9.6 [8.5–10.8]	0.1	9.0 [8.6–9.4]
**Duration of hospitalization**^e^	4 [3–10]^a^	2 [1–4]	2 [1–4]	4 [3–7]	0.006	2 [1–4]
**Deaths**	10 (66.6)^c^	6 (6.0)^c^	3 (1.4)	2 (5.7)	<0.001	21 (6.0)
Death within 24 hr	3 (20.0)^c^	5 (5.0)^b^	1 (0.5)	0	<0.001	9 (2.5)
Time to death (days)	3.9 (1.2–6.5)	1.6 (0–3.9)	3.6 (0–13.1)	1.5 (0–20)	0.5	3.0 (1.6–4.3)
Age at death (months)	35.9 (7.2–64.5)^b^	46.6 (5.4–87.7)		52.2 (49.8–154.2)	0.6	49.7 (28.6–70.9)

Numbers and (percentages). Median and (interquartile range), Mean and [95% confidence interval]

^a^IMCI: integrated management of childhood illness

^b^p ≤0.05 when compared to euglycemia

^c^p ≤ 0.001 when compared to euglycemia (details are provided in the text)

^d^Weight for height or height for age or weight for age or BMI ≤ 3 Standard Deviation (WHO 2006)

^e^days of hospitalization for survivors.

Of 350 children, 62.2% (n = 218) were severely ill, and 49.1% (n = 172) had at least one IMCI danger sign. A total of 15 (4.2%, 95% CI: 2.4–6.9) had hypoglycemia, 99 (28.2%, 95% CI: 23.6–33.3) low glycemia, 201 (57.4%, 95% CI: 52.0–62.6) euglycemia and 35 (10.0%, 95% CI: 7.0–13.6) hyperglycemia. Overall 149 (42.5%, 95% CI: 37.3–47.9) children presented with abnormal blood glucose. The distribution of blood glucose was skewed: median glycemia 5.2 mmol/L (IQR: 5.0–5.4) (Shapiro-Wilk test p < 0.001).

Children with hypoglycemia and hyperglycemia were younger than those with euglycemia. Hypoglycemia tended to be associated with being a female (p = 0.07) and belonging to the poorest families (p = 0.01) with the highest debts (p = 0.002). Hypoglycemia was associated with longer fasting (p = 0.001) and fewer immunizations (p = 0.05). Compared to euglycemia children, hypoglycemia was associated with mothers of lower educational levels (p = 0.03), lower family income and lower daily budget (p<0.001), increased frequency of debts (p = 0.001) and poverty (p = 0.03). Children with hypoglycemia had more frequently fasted longer than 12 hours (p<0.001) and a severe IMCI score (p = 0.02). More often than euglycemic children, they had at least one danger sign (p<0.001) including inability to drink (p<001), being unconscious (p = 0.002), vomiting (p = 0.004), having diarrhea (p = 0.002) or convulsions (p<0.001). Similarly, they had more often severe dehydration (p<0.001), a trend to severe malnutrition (p = 0.07) and a decreased oxygen saturation (p = 0.009). They stayed longer at the hospital, had the highest fatality rate (CRF: 66.6%; OR: 132; 95%CI: 29.0–596) and highest early death rate (death within 24 hours: 20%, p<0.001).

Compared to euglycemic, hyperglycemic children were younger, had less frequently received a treatment before admission (p = 0.01), presented with more frequent severe IMCI score (p = 0.001), severe pneumonia (OR: 21.7; 95%CI: 11.7–39.8), severe malnutrition (p = 0.01) and decreased oxymetry (p = 0.001).

Low glycemia was not associated with any specific socio-economic characteristics. Compared to euglycemia, low glycemia was associated with more frequent vomiting (p = 0.002), acute diarrhea, severe and moderate dehydration (p<0.001), and decreased oxymetry (SaO2) (p = 0.02). Low glycemic children had a higher rate of early death (5%, p = 0.008) and a fourfold risk of death compared to euglycemic children (p = 0.02).

A total of 21 (6.0%) of the children died including 9 (2.5%) during the first 24 hours of admission and 5 (1.4%) children who died within 3 days of hospital discharge Table 3. Median baseline blood glucose was lower in fatalities than survivors (2.5 mmol/dl, IQR: 2.1–4.3 and 4.7 mmol/dl, IQR: 4.3–5.8, respectively; p<0.001).

The univariate analyses of factors associated with deaths is shown Table 4. The following were factors associated with an increased risk of death: prolonged fasting over 5 hours, having an illiterate or young mother and living in poverty, absence of immunizations, the presence of danger signs according to IMCI screening: inability to drink, severe dehydration, severe malnutrition and coma, hypoglycemia, low glycemia, hepatomegaly (or enlarged liver) and having hypoxemia (oxygen saturation below 90%).

**Table 4:** Characteristics associated with death for children on admission to Vientiane hospital (univariate analysis).

	Mortality				
	n	(%)	Crude OR	95%CI	p
**Euglycemia**	3/201	1.4	1 (Ref.)		
Hypoglycemia	10/15	66.6	132	29.0–596.5	<0.001
Low glycemia	6/99	6.0	4.2	1.1–15.6	0.02
Hyperglycemia	2/35	5.7	4	0–21.9	0.1
Abnormal blood glucose	18/149	12.1	9.0	2.7–29.3	<0.001
**Socio-characteristics**					
Male	5/138	3.6	1 (Ref.)	1	
Female	16/212	7.5	2.1	0.8–5.8	0.1
Aged ≥12 Months	14/246	5.7	1 (Ref.)		
Aged <12 Months	7/104	6.7	1.1	0.4–2.9	0.8
Some immunization	13/310	4.2	1 (Ref.)		
Never immunized	8/40	20.0	5.7	2.2–14.1	0.001
Urban residential area	5/125	4.0	1 (Ref.)		
Rural residential area	16/225	7.1	1.8	0.6–4.9	0.3
Not poor	2/136	1.5	1 (Ref.)		
Poor	19/214	8.9	6.5	1.7–23.8	0.004
Mother ≥25 years	84/329	25.5	1 (Ref.)		
Mother <25 years	11/21	52.4	3.2	1.3–7.6	0.007
**Education (Mother)**					
Tertiary	1/105	0.9	1 (Ref.)		
Illiterate	4/7	57.1	138.6	14.7–256.2	<0.001
Primary	10/99	10.1	11.6	1.8–62.1	
Secondary	6/135	4.4	4.8	0.6–33.7	
**Disease Characteristics**					
Direct admission	18 /327	5.5	1 (Ref.)		
Referral	3/23	13.0	2.5	0.7–9.0	0.14
Illness ≥2 days	1/33	3.0	1 (Ref.)		
Illness <2 days	20/317	6.3	2.1	0.06–3.4	0.4
Fasting < 5 hrs	51/329	15.5	1 (Ref.)		
Fasting ≥5 hrs	10/21	47.6	4.9	2.0–12.0	<0.001
Conscious	14/317	4.4	1 (Ref.)		
Coma	7/33	21.2	5.8	2.3–14.2	0.002
Able to drink	14/328	4.2	1 (Ref.)		
Unable to drink	7/22	31.8	10.4	3.6–29.7	<0.001
Acute diarrhea	6/62	9.6	1.9	0.7–5.2	0.1
No dehydration	13/294	4.4	1 (Ref.)		
Severe dehydration	5/12	41.6	15.4	4.5–53.0	<0.001
No malnutrition	15/304	4.9	1 (Ref.)		
Severe malnutrition^a^	6/46	13.0	2.8	0.8–8.4	<0.03
No pneumonia	16/289	5.5	1 (Ref.)		
Severe pneumonia	5/61	8.2	1.5	0.5–4.3	0.4
No vomiting	11/202	5.4	1 (Ref.)		
Vomiting	10/148	6.7	1.2	0.5–3.0	0.6
No convulsion	19/317	5.9	1 (Ref.)		
Convulsion	2/33	6.0	1.0	0.2–4.5	0.9
No hepatomegaly	10/239	4.1	1 (Ref.)		
Hepatomegaly	11/111	9.9	2.5	1.0–5.9	0.03
SaO2≥90	13/302	4.3	1 (Ref.)		
SaO2<90	8/48	16.6	4.4	1.7–11.3	0.002

OR: Odd ratio; CI: Confidence interval. Variables with p<0.2 were included in the multivariate analysis of Table 5.

^a^Weight for height or height for age or weight for age or BMI ≤ 3 Standard Deviation

In multivariate analyses, hypoglycemia (OR: 197; 95%CI: 33–1173.9), decreased SaO2 (OR: 5.3; 95%CI: 1.4–20.0), presence of hepatomegaly (OR: 8.7; 95%CI: 2.0–37.6) and having an illiterate mother (OR: 25.9; 95%CI: 4.2–160.6) were features associated with increased risk of death Table 5. Similar results were obtained when including the number of danger signs into the model (Data not shown).

**Table 5:** Multivariate analysis of the characteristics associated with death for children on admission to Vientiane hospital. Only significative variables are presented.

	Mortality				
	n	(%)	OR. Adj.	95%CI	p
Euglycemia	3/201	1.5			
Hypoglycemia	10/15	66.6	197	33–1173.9	<0.001
Low glycemia	6/99	6.1	2.3	0.4–14.0	0.366
Hyperglycemia	2/35	5.7	1.9	0.2–18.9	0.591
Education (Mother)					
Literate	3/243	1.2			
Illiterate	4/7	57.1	25.9	4.2–160.6	<0.001
Disease characteristics					
No hepatomegaly	10/239	4.1			
Hepatomegaly	11/111	9.9	8.7	2.0–37.6	0.004
SaO2≤90	13/302	4.3			
SaO2 <90	8/48	16.7	5.3	1.4–20.0	0.012

OR Adj.: Adjusted odd ratio; CI: Confidence interval.

## Discussion

Our findings of this prospective study show a high frequency of abnormal blood glucose concentration [149/350 (42.5%; 95% CI: 37.3–47.9)] and a high case fatality rate (CFR) [12.0%, 95% CI: 7.3–18.4)] among ill non-neonate children attending the admission unit of a referral hospital in Laos. These abnormal blood glucose concentrations included low glycemia (28.2%), hyperglycemia (10.0%) and hypoglycemia (4.2%). Cut-offs were chosen similar to the ones used in Mali and Madagascar studies to allow comparison [13,28]. Hypoglycemia and hyperglycemia rate were similar to our previous study in Madagascar [13]. Our studies confirm hypoglycemia as a major factor associated with increased risk of death in children living in non malaria settings, despite a lower frequency than in malaria areas [12,20, 22,46,54–55]. We also found that a low oxygen saturation (<90%) and enlarged liver are potential predictors of poor prognosis [23].

Low glycemia raises various concerns. Similar to our previous study in Madagascar, low glycemia was the most common glycemic dysregulation. Low glycemia children had 4.2 fold increased risk of death in univariate analysis but this link was not observed after multivariate analysis. A possible reason was the small number of children with hypoglycemia. CFR in low glycemia children was similar to that observed in Madagascar (CFR: 5.9%, OR: 11.7, 95%CI: 4.2–32.4) and in febrile children of Tanzania using a close 2.5–5 mmol/L cut off (CFR: 7.3%; OR: 3.0, 95%CI: 2.1–4.2) [13,17]. Nevertheless, the reason explaining the mortality excess in children with low glycemia remains unclear. It is possible that this increased risk of mortality simply reflects the increased occurrence of severe hypoglycemia later in the admission and/or its association with malnutrition. To date, no studies have reported the relationship between the evolution in time of low glycemia and mortality, which implies the need for additional studies.

The WHO guidelines currently defines hypoglycemia as blood glucose <2.5 mmol/L in a sick child without severe malnutrition [42]. There is an increasing amount of evidence, particularly in malaria areas, which suggest increasing the blood glucose cut-off for administering glucose in sick children [14,17,28]. Our paper provided another contribution to this assumption for children living in developing countries outside of malaria areas.

In order to improve the poor outcomes linked to hypoglycemia, it has been recommended to start, as soon as possible, an effective treatment adapted to local resources. Such care should be widely available whether it facilitates a quicker hospital discharge or effectively limits the excess mortality. However, there is not yet enough evidence on the clinical impact of the treatment of low glycemia. Additional studies are required to establish when and how to best treat the low glycemia in sick children.

The mechanism of hypoglycemia is related with a limited tolerance for fasting due to limited glycogen storage capability and endogenous glucose production [4]. Therefore, children may only be able to maintain a normal plasma glucose level for a fasting period of no more than 12 hours [56]. The onset of hypoglycemia may be directly promoted by fuel imbalance, like a gradual decrease in availability of glucose and free fatty acids. Similar to our previous study in a non malaria area of Madagascar, the occurrence of hypoglycemia and the risk of mortality are linked to both duration of fasting and malnutrition status [57].

The maintenance of normal blood sugar concentration is also dependent upon functionally intact glycogenolytic and gluconeogenic enzyme systems. We found a strong association between hypoglycemia and hepatomegaly in our non malaria children that may be linked to the failure of hepatic regulation. On the other hand, hepatomegaly, a common feature of cardiac failure in children, was demonstrated to be independent risk factor for death among children from rural communities with infectious diseases [58], cardiomyopathy [59] or moderate to severe malnutrition [60].

Finally, as noted in previous studies, hypoglycemia was strongly associated with a deep breathing and hypoxemia reflecting hyperlactatemia closely related to unfavorable prognosis [23].

New commercially available bedside diagnostic tools to measure blood sugar levels may expedite clinical decision-making in the management of critically ill children in resource-constrained settings. Depending on the local costs and availability of the tests, their use is most commonly limited to children with altered conscious level. Broadening their recommendation to children with deep breathing and hepatomegaly would improve the diagnosis of children with hypoglycemia and may potentially improve their outcomes.

The 2013 WHO guideline recommends a systematic screening of severely ill children for hypoglycemia and the treatment by administering intravenous dextrose in the unconscious child as a first strategy [42]. This is often not feasible in low resources settings where trained personnel and essential supplies are not available. Alternatively, the WHO guideline recommends 50 ml of 10% glucose or sucrose solution (one rounded teaspoon of sugar in three tablespoons of water) orally or by nasogastric tube, followed by the first feed as soon as possible. However nasogastric tube insertion remains a difficult challenge for unskilled trained staff lacking of equipment. The third strategy, intra-osseous glucose administration, may not be practical in areas where hygiene and technical skills are still problematic. In response to this drawback, we demonstrated the feasibility and efficacy of sublingual sucrose in two studies, the first in uncomplicated malaria and the other one in severe malaria [46,48]. In remote health facilities, there is often a very narrow window of time, to save a child’s life. Therefore, we recommend the provision of such simple techniques to quickly raise the blood sugar level.

Hyperglycemia prevalence (10%) was similar to that observed in Madagascar (11%, RR: 2.2, 95% CI: 1–4.7) [13]. In our study, hyperglycemia was not associated with an increased mortality risk in univariate analysis as in Madagascar [13] or with increased convulsions as in Mali [46]. This may be due to the limited sample size (n = 35) of hyperglycemic patients, that should have been twice larger to evaluate this association.

We found a direct relationship between mortality and income of the family as well as the level of the mother’s education. This confirms our previous study in Madagascar emphasizing the impact of family poverty on the occurrence of blood glucose disturbance and mortality rate. In this sense, we emphasize again on the persistent issue for advocating improved home care, family education about the care of sick children, including the continuous feeding of sick children.

Nine of the 21 deaths (42.8%) after inclusion took place during the first 24 hours and 5 (1.4%) deaths had occurred within 3 days of hospital discharge when a systematic call of parents was conducted. Early death rate was up to 50% and 87% in our previous studies in Madagascar and Mali, respectively [13,28]. This remains a common feature of tropical pediatric facilities, as many children present with severe and advanced disease. At the health care unit level, triage facilities, availability of emergency medications, rapid diagnostic tools and capacity about initiation of resuscitative have to be improved [42].

The systematic search of post-hospital discharge deaths is not frequently conducted. Our study revealed that nearly a quarter of fatalities associated with glycemia disturbances would have been missed without follow-up phone calls to learn about the children’s status after discharge. It is common practice in Lao PDR to send patients home for humanitarian reasons or to limit the financial consequences of a death at the hospital for the families.

### Limitations of the study

This study was performed in field conditions within a busy pediatric ward of a university hospital. This excluded linking clinical outcome to blood glucose levels measured in children over time. The number of children who died before admission was not assessed. This may have underestimated the rate of hypoglycemia and may partially explain the apparent differences with other studies. The small sample size of the study probably explains why no death association with low glycemia was seen after multivariate analysis. This point requires that larger studies address the question. Additional limitations are the limited sample size of patients and the exclusion of surgical cases.

Comparison with other studies is hampered by the various thresholds for hypo and hyperglycemia definitions. To limit this, we used similar thresholds as in our previous papers.

Validity of the rapid glucose test for the low threshold has been questioned and may lead to potential underestimation [61]. To limit this bias, we systematically checked blood glucose concentrations on 1/3 sample. Of 123 intravenous samples, all but three measures (97.5%) were concordant: 2 had a minor difference (0.4 and 0.8 mmol/L) and one measure was wrong, 2 mmol/L instead of 10 mmol/L suggesting glucose administration during the time interval.

## Conclusion

Hypoglycemia and abnormal blood glucose are associated with a high risk of mortality for non neonate children in non malaria tropical settings. These results should encourage advocacy for improvements of local health facilities, especially pre-hospital care and management, which could impact the CFR. Emergency care at the district level could be improved with rapid tests for hypoglycemia. Quick and easy methods for glucose administration such as the sublingual route should be developed. International recommendations and support are needed for implementation at the district level in resource limited countries. Specific attention must be paid to both low- and hyper-glycemia associated with higher risk of death than euglycemia. Low glycemia may be considered as a threshold for treatment of children living in resource-poor settings and further evaluations of intervention using thresholds including low glycemia are recommended in resource-limited settings.

## Supporting Information

S1 Dataset (XLSX)
